# A 5-year clinical follow-up study from the Italian National Registry for FSHD

**DOI:** 10.1007/s00415-020-10144-7

**Published:** 2020-08-19

**Authors:** Liliana Vercelli, Fabiano Mele, Lucia Ruggiero, Francesco Sera, Silvia Tripodi, Giulia Ricci, Antonio Vallarola, Luisa Villa, Monica Govi, Louise Maranda, Antonio Di Muzio, Marina Scarlato, Elisabetta Bucci, Lorenzo Maggi, Carmelo Rodolico, Maurizio Moggio, Massimiliano Filosto, Giovanni Antonini, Stefano Previtali, Corrado Angelini, Angela Berardinelli, Elena Pegoraro, Gabriele Siciliano, Giuliano Tomelleri, Lucio Santoro, Tiziana Mongini, Rossella Tupler

**Affiliations:** 1 Department of Neurosciences “Rita Levi Montalcini”, Center for Neuromuscular Diseases, University of Turin, Turin, Italy; 2 Department of Life Sciences, University of Modena and Reggio Emilia, Modena, Italy; 3 Department of Neurosciences, Reproductive and Odontostomatological Sciences, University Federico II of Naples, Naples, Italy; 4 Department of Public Health, Environments and Society, London School of Hygiene and Tropical Medicine, London, UK; 5 Department of Neurosciences, University of Padua, Padua, Italy; 6 Department of Clinical and Experimental Medicine, Neurological Clinic, University of Pisa, Pisa, Italy; 7 Department of Biomedical, Metabolic and Neural Sciences, University of Modena and Reggio Emilia, via G. Campi 287, 41125 Modena, Italy; 8 Center for Neuroscience and Neurotechnology, University of Modena and Reggio Emilia, Modena, Italy; 9 Neuromuscular Unit, Fondazione IRCCS Ca’ Granda Ospedale Maggiore Policlinico, Dino Ferrari Center, University of Milan, Milan, Italy; 10 Department of Population and Quantitative Health Sciences, University of Massachusetts Medical School, Worcester, USA; 11 Center for Neuromuscular Disease, CeSI, University “G. D’Annunzio”, Chieti, Italy; 12 INSPE and Division of Neuroscience, IRCCS San Raffaele Scientific Institute, Milan, Italy; 13 Department of Neuroscience, Mental Health and Sensory Organs, S. Andrea Hospital, University of Rome “La Sapienza”, Rome, Italy; 14 IRCCS Foundation, C. Besta Neurological Institute, Milan, Italy; 15 Department of Neurosciences, Policlinico “G. Martino”, University of Messina, Messina, Italy; 16 Neurology Clinic, Spedali Civili Hospital, Brescia, Italy; 17 IRCCS San Camillo, Venice, Italy; 18 Unit of Child Neurology and Psychiatry, IRCCS “C. Mondino” Foundation, Pavia, Italy; 19 Department of Molecular Cell and Cancer Biology, University of Massachusetts Medical School, Worcester, USA; 20 Li Weibo Institute for Rare Diseases Research at the University of Massachusetts Medical School, Worcester, USA

**Keywords:** FSHD, D4Z4 reduced allele, Clinical categories, Follow-up

## Abstract

**Background:**

The natural history of facioscapulohumeral muscular dystrophy (FSHD) is undefined.

**Methods:**

An observational cohort study was conducted in 246 FSHD1 patients. We split the analysis between index cases and carrier relatives and we classified all patients using the Comprehensive Clinical Evaluation Form (CCEF). The disease progression was measured as a variation of the FSHD score performed at baseline and at the end of 5-year follow-up (ΔFSHD score).

**Findings:**

Disease worsened in 79.4% (112/141) of index cases versus 38.1% (40/105) of carrier relatives and advanced more rapidly in index cases (ΔFSHD score 2.3 versus 1.2). The 79.1% (38/48) of asymptomatic carriers remained asymptomatic. The highest ΔFSHD score (1.7) was found in subject with facial and scapular weakness at baseline (category A), whereas in subjects with incomplete phenotype (facial or scapular weakness, category B) had lower ΔFSHD score (0.6) *p* < 0.0001.

**Conclusions:**

The progression of disease is different between index cases and carrier relatives and the assessment of the CCEF categories has strong prognostic effect in FSHD1 patients.

**Electronic supplementary material:**

The online version of this article (10.1007/s00415-020-10144-7) contains supplementary material, which is available to authorized users.

## Introduction

Facioscapulohumeral muscular dystrophy (FSHD, OMIM # 158900) is a hereditary myopathy with prevalence of 1 in 8500–20,000 individuals [1, 2]. The classical FSHD phenotype is characterized by a distinctive distribution of muscular weakness [3].

Two genetically distinct disease subtypes, FSHD1 and FSHD2 have been described. The vast majority of FSHD subjects, named FSHD1, carry contractions of a polymorphic tandemly arrayed 3.3 kb D4Z4 repeat element on the telomeric region of chromosome 4, at 4q35 [4]. Detection of one D4Z4 alleles with 10 or fewer repeats associated with the 4qA polymorphism is considered a molecular hallmark for FSHD diagnosis [5]. FSHD2, which represents 5–10% of cases, is contraction-independent, with affected individuals carrying two D4Z4 arrays in the healthy range (> 10 RUs) [6].

Since the discovery of the D4Z4 locus for FSHD diagnosis it was clear that many different phenotypes and reduced penetrance [7–17] can be observed in people carrying a D4Z4 reduced allele (DRA). These features have substantially hindered the possibility of defining the progression modes and the natural history of FSHD [18, 19] with critical consequences on clinical management. The few studies that attempted the description of the FSHD natural history confirmed the considerable variability with several difficulties to identify a marker that may serve as predictor of decline [16, 18–20].

In previous studies we designed the FSHD clinical score, a tool to capture the degree of clinical disability, and the Comprehensive Clinical Evaluation Form (CCEF) for the standardized description of clinical phenotypes [21, 22]. Recently, through the use of the CCEF we clarified that there is a clinical phenotypic spectrum in molecularly homogeneous genetic subgroups. In particular, carriers of 7–8 DRA, until now considered in the classical FSHD range, present a clinical variability that is quite similar to that found among subjects carrying one 9 -10 DRA [9], which are instead considered borderline alleles [11]. Some genotype–phenotype studies suggest carriers 7–10 DRA have a low penetrance and, in this subgroup, the muscular impairment of carriers relatives is less severe than index cases [9, 17, 20]. By contrast carriers of 1–3 DRA present less significant differences [8]. Moreover, subjects with a facial and scapular involvement are more severely affected than subjects with facial sparing myopathy [23, 24]. All these observations suggest that disease progression can differ on the basis of size of DRA, degree of kindship or phenotypic features.

Here we report the results of a multi-centric longitudinal cohort study of 246 subjects from the Italian National Registry for FSHD (INRF) database. We reviewed the phenotypic characteristics of index cases and carrier relatives carrying one DRA within the size range of 1–10 Repeat Units (RUs) at baseline and after 5-year follow-up. To model the long-term disease progression, we analyzed how sex, DRA size, age at onset, disease duration, and clinical phenotype affect the progression rate in index cases and carrier relatives.

## Methods

### Study design and participants

Our multi-centric longitudinal cohort study was performed in 14 Italian FSHD-experienced centers of the Italian Clinical Network for FSHD (ICNF). 246 Caucasian individuals (141 index cases and 105 carrier relatives from 63 family) from a consecutive group were enrolled between January 10th, 2007 and December 20th, 2011 for the baseline visit. All individuals included in this study carry one DRA within the size range of 1–10 repeat units (RU) associated with the permissive haplotype 4qA. We considered a follow-up period of 5 years; therefore, the last visit was performed between February, 2012 and December, 2016. We enrolled only patients for which the last clinical evaluation has been performed using the CCEF, applied by a properly trained physician of the INCF. The 5-year time of the clinical follow-up was considered a significant period in which the disease may evolve or appear in healthy carrier relatives. In ten out of 14 centers, individuals were evaluated by the same investigators at all visits, whereas in the remaining centers evaluators changed for a subset of patients.

### Clinical investigation

The Comprehensive Clinical Evaluation Form (CCEF) [21] was used to classify all DRA carriers. The CCEF divides the carriers as following: (1) individuals presenting facial and scapular girdle muscle weakness typical of FSHD (category A, subcategories A1–A3), (2) individuals with muscle weakness limited to scapular girdle or facial muscles (category B subcategories B1, B2), (3) asymptomatic/healthy individuals (category C, subcategories C1, C2), (4) individuals with myopathic phenotype presenting clinical features not consistent with FSHD canonical phenotype (D, subcategories D1, D2).

As primary outcome measure, we used the ΔFSHD score obtained by comparing the FSHD score at baseline and at follow-up. Disease progression was assessed as increment of the FSHD score [22]. The FSHD score ranges from 0, when no objective sign of functional impairment is present, to 15, when all tested muscle groups are severely impaired and patient is wheel-chair dependent (see https://www.fshd.it for training). The evaluation protocol is specifically designed for FSHD. Each section describes the functional evaluation of six muscle districts peculiarly affected in FSHD: face (score 0–2); shoulder girdle (score 0–3); upper limbs (score 0–2); distal legs (score 0–2); pelvic girdle (score 0–5); abdominal muscles (score 0–1). Diversely from the commonly used Clinical Severity Scale (CSS) [10], this protocol attributes an independent score to each distinct muscle group thus providing an accurate description of the distribution of muscle weakness for each individual.

In addition, we evaluated the strength of ten different muscle groups on both right and left side using the Medical Research Council (MRC) grading scale (0–5) [25–28]: forearm flexor/extensor muscles, hand and wrist flexor/extensor muscles, thigh flexor, knee extensor, and foot extensor/flexor muscles. The MRC evaluation was carried out by a neurologist previously trained in clinical trials using this methodology [29]. All tests and evaluations were performed in a blind manner with respect to the results of the D4Z4molecular analysis. A very good inter-rater reliability of assessment has been shown in our previous studies testing our clinical evaluation methodology [21, 22].

Age at onset and the first muscle group affected by disease were derived from patients’ records or recollections [30]. Individuals were asked some questions to retrieve more accurate information that have been proved relevant or indicative for FSHD.

The INRF database was approved by the ethics committee of the Province of Modena. Informed written consent was obtained from all study participants, in accordance with the ethical standards of the 1964 Declaration of Helsinki.

### Molecular characterization

DNA was prepared from isolated lymphocytes according to standard procedures. Restriction endonuclease digestion of DNA was performed with the appropriate restriction enzyme: EcoRI, EcoRI/BlnI. Digested DNA was separated by pulsed field gel electrophoresis (PFGE) in 1% agarose gels, as previously described [31] and by linear 0.4% gel electrophoresis. Allele sizes and the presence or absence of the 4qA allele were estimated by Southern hybridization with probes p13E-11 and 4qA, respectively, run with High Molecular Weight Marker and 2.5 kb DNA ladder. Restriction fragments were detected by autoradiography.

### Statistical analysis

Baseline characteristics of the study cohort for index cases and carrier relatives were summarized with mean and standard deviation for quantitative variables and frequencies distribution for qualitative variables. To evaluate differences between index cases and carrier relatives with respect to quantitative variables we used the t test, while chi-square test was used to evaluate whether the distribution of qualitative variables was similar in index cases and carrier relatives. Same tests were used to compare quantitative and categorical variables on females and males. We used one-way analysis of variance (ANOVA) to evaluate whether size of the DRA or CCEF clinical classification were associated with FSHD and ΔFSHD score. The ANOVA was also used to evaluate the associations between age at onset and FSHD score and ΔFSHD score. To evaluate the impendent association of the size of the DRA and CCEF clinical classification with ΔFSHD score a multivariable regression model was fitted adjusting for age, sex, FSHD score at baseline and length of follow-up. Missing values were not imputed.

### Data availability

The data that support the findings of this study are available upon request at miogenlab@unimore.it.

## Results

### General findings

The study population consisted of 246 individuals carrying one DRA, 141 index cases, 84 (59.6%) males, and 105 carrier relatives from 63 families, 52 (49.5%) males. Demographics, molecular and clinical data are given in Table 1. At baseline, the average age of index cases was 46.1 ± 14.2 years; that of carrier relatives was 38.3 ± 15.6 years. The average duration of follow-up of index cases was 6.1 ± 1.2 years; that of carrier relatives was 5.8 ± 0.9 years.

**Table 1:** Cohort characteristics at baseline

	Index cases (*n* = 141)	Carrier relatives (*n* = 105)
	*n* (%)	*n* (%)
Men	84 (59.6)	52 (49.5)
	Mean (SD)	Mean (SD)
Age (years)	46.1 (14.2)	38.3 (15.6)
Age at onset (years)	25.7 (14.8)	29.0 (16.5)^a^
Disease duration (years)	20.5 (14.3)	10.0 (11.5)^a^
D4Z4 allele size (RU)	*n* (%)	*n* (%)
1–3	12 (8.5)	4 (3.8)
4–5	37 (26.2)	26 (24.8)
6	41 (29.1)	39 (37.1)
7–8	29 (26.6)	16 (15.2)
9–10	22 (15.6)	20 (19.1)
FSHD score	*n* (%)	*n* (%)
0	1 (0.8)	47 (44.8)
1–2	18 (12.8)	31 (29.5)
3–7	75 (53.2)	25 (23.8)
8–15	47 (33.3)	2 (1.9)

^a^Calculated on 58 carrier relatives with FSHD symptoms at baseline examination

The duration of disease (calculated from the onset of the first symptoms to the first examination at time 0) varies between 0 and 54 years (mean time 20.5 ± 14.3 years) for index cases and between 0 and 41 years (mean time 10.0 ± 11.5 years) for carrier relatives.

At onset, 133 individuals (66.8%) reported weakness of scapular girdle muscles, 23 (11.6%) reported facial muscle weakness, 6 (3.0%) pelvic girdle weakness, 26 (13.1%) lower limb involvement (foot drop). Eleven patients (5.5%) did not report any symptom but at their first examination seven presented mild facial weakness, one had isolated scapular girdle weakness, two suffered from scapular girdle weakness associated with lower limb involvement. Age at onset was significantly different between females and males (29.6 ± 14.3, and 24.2 ± 16.4, respectively; *p* = 0.014). At baseline, three individuals (1.2%) were not ambulant; one individual (0.4%) was on non-invasive ventilation (NIV). At the end of follow-up period seven index cases (5%) and one relative (1%) lost ambulation and five index cases had started NIV (3.3%).

### FSHD score at baseline

Disease severity was defined by the FSHD score. At baseline, in index cases the mean FSHD score was 6.3 ± 3.3, in carrier relatives 1.8 ± 2.5. As shown in Table 1, at baseline one index case out of 141 (0.8%) showed slight increase (2 ×) of blood creatine kinase, diffuse myalgias and early fatigability without functional motor impairment, 18 index cases (12.8%) were minimally affected (FSHD score 1–2); 75 (53.2%) were moderately affected (FSHD score 3–7); 47 (33.3%) were severely affected (FSHD score 8–15). Among the 105 carrier relatives, 47 (44.8%) were healthy (FSHD score 0); 31 (29.5%) were minimally affected individuals; 25 (23.8%) were moderately affected, two (1.9%) were severely affected.

### Variation of the FSHD score at follow-up (ΔFSHD score)

**Fig. 1:**
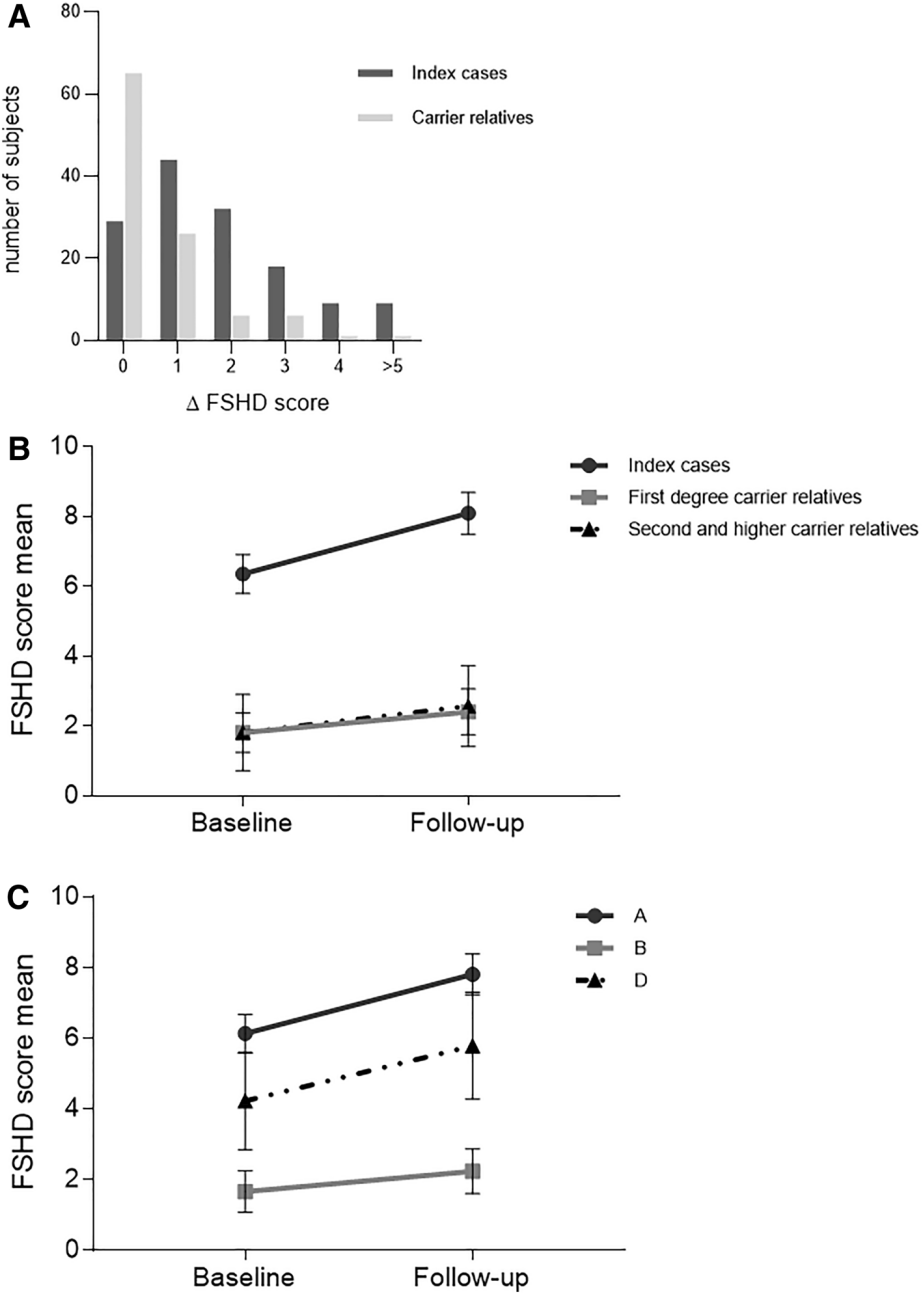
**a** Distribution Δ FSHD score of between index cases and carriers relatives. The percentage of index cases or carriers relatives with 5 different sets of Δ FSHD score is presented. **b** Proposed disease trajectories of index cases and carriers relatives. These trajectories describe the functional decline measured by the FSHD score in index cases and carriers relatives. Index cases received a higher FSHD score at baseline than carriers relatives. The ΔFSHD score is higher in index cases than carriers relatives. Data are represented as mean ± CI. **c** Proposed disease trajectories of clinical category. The trajectories describe the functional decline measured by the FSHD score in clinical category A, B and D. Data are represented as mean ± CI

We evaluated the extent of disease progression, measuring the increment of the FSHD score (ΔFSHD score) at follow-up. As shown Fig. 1a, the FSHD score of the first evaluation was maintained in 20.6% of index cases and 61.9% of carrier relatives. In general, in our cohort we observed 1.3 (1.1; 1.4) increase in the FSHD score at the end of follow-up period (mean FSHD score 4.4 ± 3.8 SD at baseline versus mean FSHD score 5.7 ± 4.3 SD at follow-up). The overall ΔFSHD score ranged widely between 0 and 7, median 1. When we selected only affected individuals with FSHD score 1–14 (*n* 196), eliminating the three most severely impaired cases (FSHD score 15) and the ones with FSHD score 0, we observed an average ΔFSHD score of 1.5 (1.3; 1.7) (mean FSHD score 5.4 ± 3.3 SD at baseline versus mean FSHD score 6.9 ± 3.8 SD at follow-up).

The separate evaluation of the ΔFSHD score in index cases and carrier relatives shows that the FSHD score increased of about 2 points in index cases (from 6.3 ± 3.3 SD at baseline to 8.1 ± 3.6 SD at follow-up), whereas it increased in of approximately 0.6 point among carrier relatives (from 1.8 ± 2.6 SD at baseline to 2.4 ± 2.9 SD at follow-up). We compared the slope of disease progression of the index cases’ group with that of the carrier relatives’ group. Figure 1b shows that the disease trajectory of the index cases’ group is steeper than the one of their carrier relatives (associated *p* value < 0.001). This observation is confirmed by the regression model. We also compared the ΔFSHD score observed in females and males (1.13 ± 1.24 versus 1.39 ± 1.57) and found no evidence of differences in disease progression between the two sexes.

### Correlation of ΔFSHD and the clinical category

**Fig. 2:**
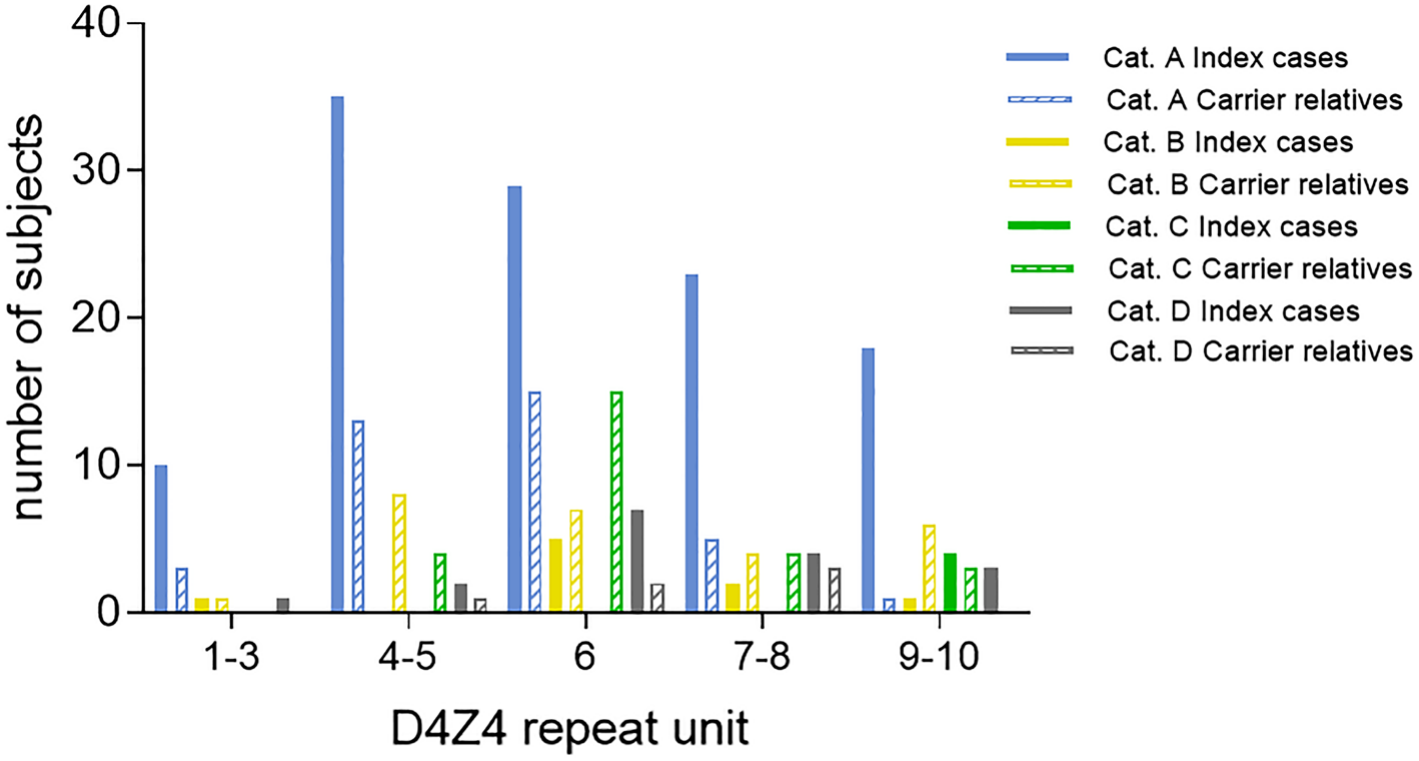
Distribution of index cases and carriers relatives carrying D4Z4 reduced allele according to D4Z4 allele size (RU) and clinical categories. Subjects were subdivided by D4Z4 reduced allele size: 1–3, 4–5, 6, 7–8, 9–10 repeat units. In each subgroup, number of index cases and carriers relatives who were assessed as clinical category A, B, C or D are reported

The distribution of clinical categories and subcategories in our cohort is shown in Supplementary Table 1: 152 (62.1%) individuals displayed the involvement of facial and scapular girdle muscles and were classified as category A. Clinical category A was much more represented in index cases than in carrier relatives [115 (81%) versus 37 (35%), respectively]. Whereas the incomplete phenotype (clinical category B) was more frequent in carrier relatives than in index cases (25% versus 6%) (*p* < 0.001). Age at onset was not significantly different between index cases and carrier relatives subdivided on the basis of the clinical subcategories (*p* = 0.5209) (Supplementary Table 1). We observed that 79.1% (38/48) of carriers without motor impairment (clinical category C) and 57.4% (27/47) of individuals with mild disability (FSHD score ≤ 2) had ΔFSHD score 0. Figure 2 shows that the distribution of clinical categories among index cases or carrier relatives is not associated with a particular size of DRA. Instead, we found that clinical category A is associated with higher FSHD score at baseline and steeper slope of disease progression (average FSHD score at baseline 6.1, ΔFSHD score 1.7) as clinical category D (average FSHD score 4.2, ΔFSHD score 1.6), whereas we observed slower disease progression in individuals with incomplete clinical phenotype (category B, average FSHD score at baseline 1.7, ΔFSHD score 0.6, *p* < 0.0001) (Fig. 1c).

### Correlation of ΔFSHD and DRA size

To estimate whether the size of DRA is a predictor of disease severity and progression, we analyzed the FSHD score and the ΔFSHD score observed in individuals carrying DRA of different size. Table 2a shows that the highest basal FSHD score was detected in the index cases carrying DRA with 1–3 RU, whereas it was lower and did not significantly vary among index cases carrying DRA with 4–10 RU. Table 2b shows that the ΔFSHD score of all index cases did not significantly vary on the basis of the DRA size. We observed that in the group carrying DRA with 4–10 RU index cases have higher ΔFSHD score than carrier relatives (*p* < 0.01), whereas we found no difference between index cases and carrier relatives carrying DRA with 1–3 RU (*p* = 0.831).

**Table 2:** Distribution of age, FSHD score (A) and Δ FSHD score (B) by D4Z4 allele size (RU) in Index cases and carrier relatives

A								
D4Z4 allele size (RU)	Index cases	Age baseline (years)	FSHD score baseline	FSHD score follow up	Carrier relatives	Age baseline (years)	FSHD score baseline	FSHD score follow up
	*n* (%)	Mean (SD)	Mean (SD)	Mean (SD)	*n* (%)	Mean (SD)	Mean (SD)	Mean (SD)
1–3	12 (75.0)	39.4 (11.6)	9.6 (2.5)	11.8 (2.8)	4 (25.0)	20.2 (16.1)	3.0 (1.4)	5.5 (3.7)
4–5	37 (58.7)	43.4 (15.8)	6.9 (3.4)	8.6 (3.5)	26 (41.3)	40.1 (18.0)	2.8 (3.7)	2.6 (2.4)
6	41 (51.3)	46.6 (14.0)	6.3 (2.9)	7.9 (3.0)	39 (48.7)	35.0 (14.9)	1.5 (2.0)	2.4 (2.6)
7–8	29 (64.1)	47.5 (13.5)	5.8 (3.4)	7.3 (3.8)	16 (35.9)	41.4 (13.6)	2.4 (2.2)	2.7 (2.2)
9–10	22 (52.4)	51.4 (13.2)	4.6 (3.2)	6.6 (3.4)	20 (47.6)	43.4 (12.8)	0.5 (1.1)	0.8 (1.4)
	Anova *F* test *p* value	0.012	0.001	0.0005		0.034	0.018	0.0043

B			
D4Z4 allele size (RU)	Δ FSHD score		
	Index cases	Carrier relatives	*t* test
	Mean (SD)	Mean (SD)	*p* value
1–3	2.25 (1.76)	2.50 (2.64)	0.831
4–5	1.78 (1.65)	0.85 (0.83)	0.010
6	1.63 (1.43)	0.56 (0.99)	0.001
7–8	1.52 (1.21)	0.31 (0.87)	0.001
9–10	1.95 (1.68)	0.35 (0.67)	0.001
Anova *F* test *p* value	0.616	0.001	

### Correlation of ΔFSHD and age at onset

We also investigated whether age at disease onset correlates with disease outcome. We considered age at examination, disease duration, FSHD score and ΔFSHD score. As shown in Table 3, in our cohort disease onset by age 10 is not associated with more severe disease outcome (*p* = 0.706).

**Table 3:** Distribution of age, disease duration, FSHD score and Δ FSHD score, by age at onset on 198 subjects with FSHD at baseline examination

Age at onset (years) (*n*)	Age baseline (years) Mean (SD)	Disease duration (years) Mean (SD)	FSHD score Mean (SD)	Δ FSHD score Mean (SD)
≤ 10 (24)	35.1 (17.6)	27.5 (17.5)	6.7 (4.2)	1.7 (2.0)
11–18 (58)	37.9 (14.7)	22.8 (14.2)	*6.2* (3.7)	1.6 (1.4)
19–35 (56)	40.6 (13.2)	15.6 (13.0)	4.9 (3.2)	1.3 (1.4)
36–54 (48)	55.0 (7.6)	10.7 (7.7)	5.1 (2.7)	1.4 (1.1)
≥ 55 (12)	61.5 (13.2)	8.8 (15.4)	3.8 (3.2)	1.6 (1.8)
Anova *F* test *p* value	*p* < 0.001	*p* < 0.001	0.024	0.706

### Evaluation of determinants of ΔFSHD score

We finally investigated the possible relationships between size of DRA, or clinical phenotype, described as clinical category, with disease progression considering age, sex, length of follow-up, and FSHD score at baseline. Table 4 shows the results of the multivariable regression models that evaluate determinants of ΔFSHD score. The multivariable models confirm the strong prognostic effect of the size of the reduced D4Z4 and CCEF categories. Interestingly, the effect is stronger on carrier relatives on which the prognostic model explains the 42% of ΔFSHD score variability. To be noted that the effect of the size of the reduced D4Z4 allele is mainly due to the difference of ΔFSHD score between carriers of 1–3 DRA versus 4–10 DRA carriers. The effect of D4Z4 size among carriers of 4–10 DRA, was not significantly different (*p* = 0.675 for index cases, *p* = 0.083 for carrier relatives). Instead, the multivariate analysis demonstrates that the effect of the size of the reduced D4Z4 allele is mainly responsible for the difference of ΔFSHD score between carriers of 1–3 DRA versus 4–10 DRA carriers.

**Table 4:** Multivariable regression models to evaluate determinants of Δ FSHD score

	All cohort		Index cases				Carrier relatives	
	Beta^a^	95% CI^c^	Beta^a^			95% CI^c^	Beta^a^	95% CI^c^
D4Z4 allele size (RU)								
Index cases	Ref^b^							
Carrier relatives	− 0.64	(− 1.07; − 0.20)						
1–3	Ref^b^		Ref^b^				Ref^b^	
4–5	− 1.05	(− 1.76; − 0.33)	− 0.97		(− 1.99; 0.05)		− 1.50	(− 2.43; − 0.56)
6	− 1.11	(− 1.82; − 0.40)	− 0.94		(− 1.97; 0.08)		− 1.68	(− 2.59; − 0.76)
7–8	− 1.42	(− 2.17; − 0.66)	− 1.24		(− 2.33; − 0.15)		− 2.12	(− 3.11; − 1.13)
9–10	− 1.07	(− 1.87; − 0.26)	− 1.05		(− 2.24; 0.15)		− 1.52	(− 2.53; − 0.52)
Clinical category								
A	Ref^b^		Ref^b^				Ref^b^	
B	− 1.45	(− 2.19; − 0.70)	–	–			− 2.25	(− 3.08; − 1.42)
C	− 0.89	(− 1.59; − 0.18)	− 0.91	(− 2.16; 0.33)			− 1.64	(− 2.45; − 0.83)
D	0.19	(− 0.39; 0.76)	0.51	(− 0.30; 1.32)			− 0.80	(− 1.58; − 0.13)
		*R*^2^ = 0.30		*R*^2^ = 0.12				*R*^2^ = 0.43

Multivariable regression models performed in the whole cohort, index cases and relatives. All the models were adjusted by age, sex, length of follow up, and FSHD score at baseline

Clinical category: (A) individuals presenting facial and scapular girdle muscle weakness typical of FSHD; (B) individuals with muscle weakness limited to scapular girdle or facial muscles; (C) asymptomatic/healthy individuals; (D) individuals with myopathic phenotype presenting clinical features not consistent with FSHD canonical phenotype

*RU* repeat unit, *R*^*2*^ coefficient of determination: % of Δ FSHD score explained by variables included in the multivariable regression model

^a^Coefficients from the multivariable regression model; they represent mean difference of the Δ FSHD score between the category and the reference level

^b^Reference level

^c^95% confidence Interval

### MRC assessment

Figure Supplementary 1A shows that at baseline, muscle strength was preserved at neurological examination (MRC grade 5/5) in all muscle groups for the large majority of 246 individuals. In particular, hand and wrist flexor/extensor muscles in 219 (89%), quadriceps in 190 (77.2%), brachialis biceps in 183 (74.4%), brachialis triceps 174 (70.7%) and tibialis anterior in 145 (58.9%).

*Tibialis anterior* and *quadriceps femoris* were significantly more affected in index cases than in carrier relatives; 35.5% of index cases versus 4.8% carrier relatives had MRC grade ≤ 3/5 of *tibialis anterior* (*p* < 0.0001 chi-square test) (Supplementary Figure 1B and 1C). In *quadriceps femoris* 63.8% of index cases and 95.2% of carrier relatives had MRC grade 5/5 (*p* < 0.0001), while 8.5% of index cases and 1.9% carrier relatives had MRC grade ≤ 3/5 (*p* = 0.027) (Supplementary Figure 1D and 1E).

At follow-up the muscle strength of *tibialis anterior* had diminished in 30% of individuals, 70 index cases (49.6%) and 22 carrier relatives (20.1%) (Supplementary Figure 1B), whereas the strength in brachialis triceps and *quadriceps femoris* muscles were reduced at a significantly lesser extent (16.3% and 5.3%, respectively).

## Discussion

FSHD is among the most common forms of muscular dystrophy with a considerable clinical heterogeneity also in genetically homogeneous cohorts [9, 11]. The study of natural history in a slowly and highly variable progressive disease such FSHD is crucial to identify sensitive, validate and reliable outcome measures in designing clinical trial. However, the natural history of FSHD has not been well defined, with most information based on historical or retrospective data. At present, only two studies describe the FSHD natural history [18, 19]. Both studies highlight the considerable variability in the progression modes among carriers of the molecular defect. The reasons for this trend are substantially unknown. Studies evaluating the modification of muscle magnetic resonance imaging (MRI) through time as possible outcome measure have not given a definitive answer [32, 33]. No definite predictors of decline of muscle strength have been identified, apart from early disease onset [34].

To our knowledge, the present work is the largest long-term clinical follow-up study in FSHD conducted on a cohort of individuals carrying D4Z4 reduced alleles.

We found that the clinical phenotype as described by the CCEF categories might be a predictor of the progression of disability with a more rapid evolution of disease in individuals presenting a classical FSHD phenotype (category A) in comparison to patients with a facial-sparing phenotype (category B1). In this respect, previous studies suggested that the facial sparing phenotype in DRA carriers may represent a different nosological entity with a mild phenotype [23, 24]. Accordingly, Mah and collaborators (2018), who studied individuals with early onset FSHD, considered that the disease has a slow progression in patients with facial sparing [16]. In the same work, the Authors concluded that earlier age at onset of facial weakness was associated with greater disease severity. These patients, in our view, correspond, in our cohort, to individuals assessed as Category A1 who displayed the most severe phenotype and accelerated disease worsening.

Notably, the identification of non-FSHD signs in DRA carriers (category D) might serve as a proxy indicator of the co-presence of other genetic defects or modulators and requires additional studies and gene testing as indicated by the numerous cases reporting the association of FSHD with other neuromuscular conditions reviewed by Refs. [35–47]. Finally, asymptomatic/healthy carriers of 4–10 RU D4Z4 alleles, classified as Category C, stay asymptomatic/healthy in 79.1% of cases over the 5-year period.

Overall, the strong prognostic effect of the clinical phenotype as described by the CCEF categories, together with the size of the DRA, is confirmed by the multivariable models considering sex, age, age at onset, disease duration, DRA size. This effect is particularly significant among carrier relatives on whom the prognostic model explains the 42% of ΔFSHD score variability.

Our data substantially confirm, in the long-term, the clinical diversity previously observed between index cases and carrier relatives [7] and show that different disease progression might be anticipated in individuals assessed as different CCEF categories. The fact that muscle impairment advances more rapidly in index cases in comparison with carrier relatives supports the notion that FSHD is a complex genetic disease with other elements, genetic and/or environmental, influencing disease progression. These results complement our earlier observation showing that the proportion of penetrance inversely correlates with the degree of kinship, 72.5% in first degree carrier relatives versus 52.9% in second/fifth degree carrier relatives [7].

Finally, the detailed investigation of muscle strength by MRC grading scale indicates that *tibialis anterior*, deteriorates at high rate in 5 years. Thus, quantifiable assessment(s) might be designed on the evaluation of this muscle to create sensitive and effective outcome measures able to detect small changes as sign of deterioration in a timeframe suitable for clinical trials.

### Limitations of the study

Our work presents methodological limitations: not using CCEF for the first-visit assessment and not evaluating patients with MRI, which is sometimes planned in medical follow-up. The mean of the disease duration of the index cases is longer than carrier relatives. This is a selection bias that could influence the clinical impairment in the index cases.

At present tools that capture the clinical progression in a short period (such as 1 or 2 years of clinical trial) are not available. In the future, other studies may be conducted with the support of clinical assessment and including other validated outcome measures, such as long-term imaging data.

### Conclusions

Our systematic study confirms the large intra-familial and inter-individual clinical variability observed in DRA carriers and demonstrates that the assessment of the CCEF categories might provide relevant information for the standardized selection of patients eligible for clinical trials and for the stratification of individuals for clinical and molecular studies.

Molecular findings seem to have a good predictive value only for individuals carrying 1–3 DRA. Instead people carrying 4–10 DRA display large clinical variability ranging from healthy carrier relatives to individuals showing complex myopathic phenotypes. This result, together with the knowledge that DRA with 4–8 RU have 3% frequency in the general population [31, 48, 49], should be considered in the guidelines for FSHD diagnosis [50].

Data reported here imply that the precise clinical description and genetic investigation are essential for the clinical management of pedigrees in which one DRA segregates. Indeed, the reduced risk of developing disease for healthy carrier relatives lessens the psychological burden of a positive molecular diagnosis and should sustain procreative decisions. It is advisable to provide genetic counseling based on clinical and molecular evaluation of each family. For people at consultation, results of molecular analyses should be considered together with the clinical categories assessed in the family members, taking into account the degree of kinship towards the index cases, as well as the penetrance observed in each individual family, whenever possible.

## Electronic supplementary material

Below is the link to the electronic supplementary material.

Supplementary file 1 (DOCX 420 kb)

## Abbreviations

CCEF: Comprehensive Clinical Evaluation Form
DRA: D4Z4 reduced alleles
FSHD: Facioscapulohumeral muscular dystrophy
INRF: Italian National Registry for FSHD
INCF: Italian National Consortium for FSHD
MRC: Medical Research Council
NIV: Non-invasive ventilation
