# Supplementary material for: A 5-year clinical follow-up study from the Italian National Registry for FSHD
Source: J Neurol. 2020 Aug 19;268(1):356–66. doi: 10.1007/s00415-020-10144-7 (PMC7815626; doi:10.1007/s00415-020-10144-7)
Supplement: Supplementary file 1 — (DOCX 420 kb) [file 415_2020_10144_MOESM1_ESM.docx]

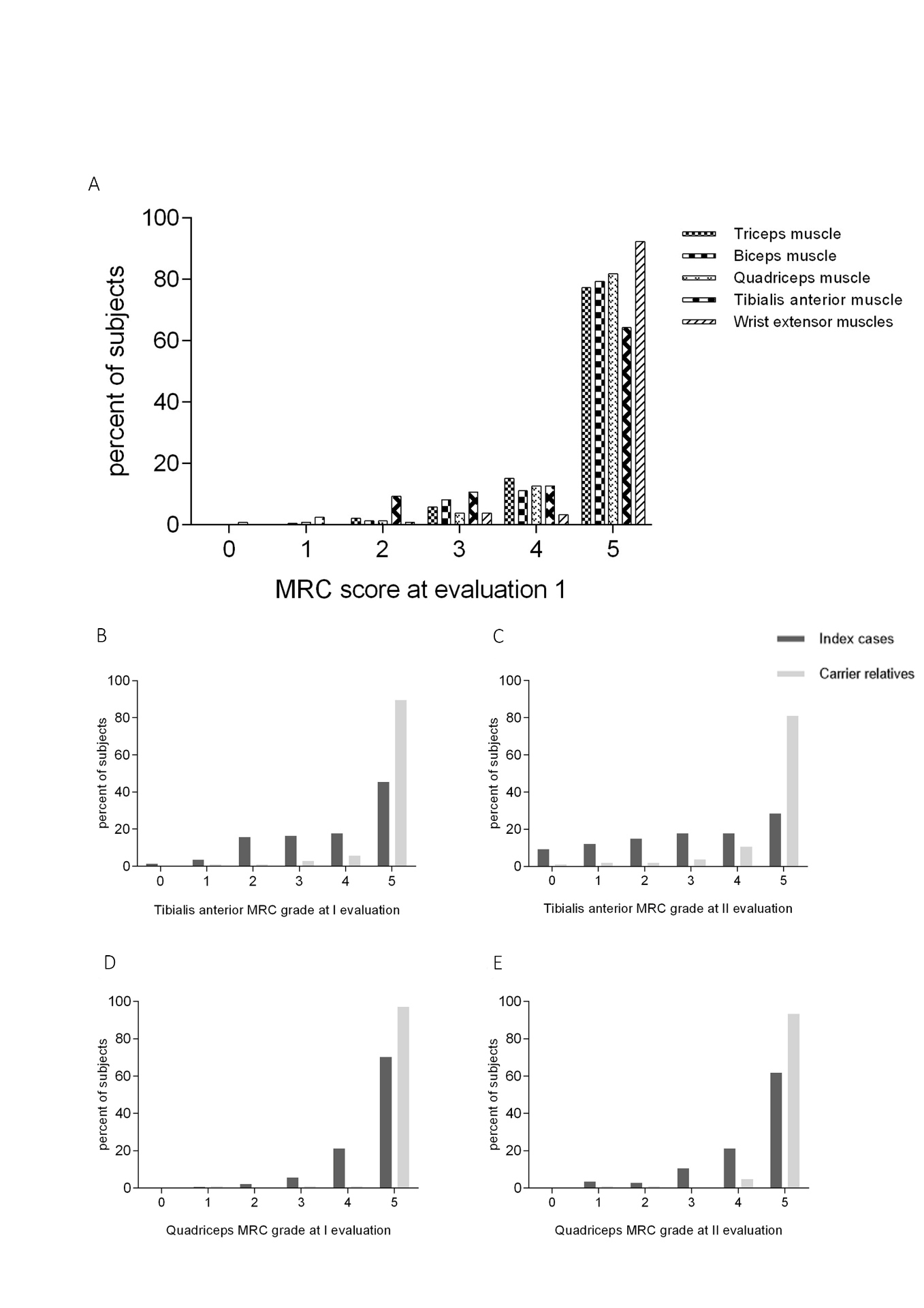


**Supplementary Figure 1.** Muscle strength of biceps, deltoid, quadriceps, tibialis, triceps in 246 subjects. MRC score was used to describe muscle weakness in the selected muscles bilaterally (A). The percentage of subjects presenting different MRC scores is reported. Muscle strength of tibialis anterior and quadriceps in Index cases and Carrier relatives. MRC score was used to describe muscle weakness of (B-C) tibialis anterior bilaterally and (D-E) quadriceps. The percentage of subjects presenting the different MRC scores is reported.
